# Acquired Hemophilia A (FVIII Deficiency) Associated with Papillary Thyroid Cancer: Treatment with Recombinant Porcine FVIII

**DOI:** 10.1155/2019/9026121

**Published:** 2019-08-28

**Authors:** S. Nguyen, P. Teh, J. Zhou, E. Y. Chang, A. von Drygalski

**Affiliations:** ^1^Department of Medicine, University of California San Diego, San Diego, CA, USA; ^2^Department of Medicine, University of California Riverside, Riverside, CA, USA; ^3^Department of Molecular Medicine, The Scripps Research Institute, La Jolla, CA, USA; ^4^Radiology Service, VA San Diego Healthcare System, San Diego, CA, USA; ^5^Department of Radiology, University of California San Diego, San Diego, CA, USA

## Abstract

Acquired hemophilia A (AHA) is a rare autoimmune disorder caused by autoantibodies against Factor VIII (FVIII). It has a high mortality due to bleeding complications. FVIIa-based bypassing agents are the first line of treatment but not always effective. Recombinant porcine (rp) FVIII (Obizur®) was recently approved for rescue treatment but with little evidence-based information regarding efficacy. We report a case of papillary thyroid cancer associated with AHA malignancy that responded to a single dose of rpFVIII after failure to achieve hemostasis with FVIIa-based bypassing products.

## 1. Case Presentation

**Figure 1:**
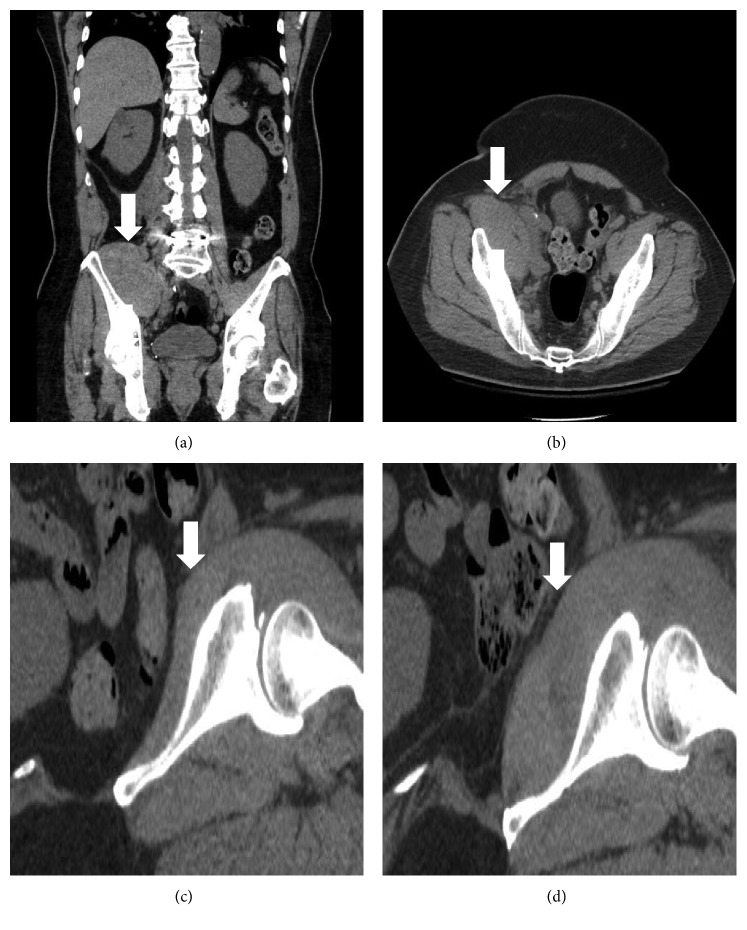
Computed tomography scan of the abdomen and pelvis. Coronal (a) and axial (b) planes show asymmetrically enlarged right iliacus muscle (white arrows), consistent with hematoma. Sagittal plane of the hips: left unaffected hip (c) in comparison to the right hip (d), which showed right psoas muscle bleed after treatment of recombinant human factor VIIa and Factor Eight Inhibitor Bypassing Activity (FEIBA).

A 71-year-old Asian male presented to the UCSD Medical Center with generalized body bruising, intermittent gingival bleeding, hematuria, and epistaxis for one month. His medical history was remarkable for metastatic papillary thyroid cancer, which was diagnosed three years earlier. He underwent total thyroidectomy, radiotherapy, and stereotactic radiosurgery and was receiving ulixertinib (BioMed Valley Discoveries, Kansas, USA), an experimental ERK1/2 kinase inhibitor, on a clinical trial protocol for patients with advanced solid tumors. The patient had no prior personal or family history of bleeding problems and was not on anticoagulation. On presentation in the Emergency Department, the patient reported acute right groin pain after a mechanical fall. His physical examination was significant for diffuse ecchymoses across the abdomen without petechiae or palpable purpura. The right hip demonstrated tenderness upon palpation. Laboratory studies showed a hemoglobin of 11.6 g/dL, normal prothrombin time (PT), prolonged activated partial prothrombin time (aPTT) (107.4 seconds) with 1% FVIII plasma activity, and a FVIII inhibitor titer of 57 Bethesda units (BU). Computed tomography (CT) scan of the abdomen showed a large right iliopsoas hematoma (Figures 1(a) and 1(b)). He was admitted and treated with rhFVIIa at 90 *μ*g·kg^−1^ every two hours and red cell transfusions during the next 2 days. However, his hemoglobin continued to drop to 7.7 g/dL. Due to unresponsiveness to rhFVIIa, the patient was switched to FEIBA (75 units/kg, 2-3 times daily) for 2 more days. His hemoglobin continued to drop to 7.4 g/dL despite blood transfusions with increasing iliopsoas bleeding on a subsequent CT scan (Figures 1(c) and 1(d)). At this point, rpFVIII at 100 units/kg was administered (half of the labeled dose), which stopped the bleeding promptly based on clinical assessment (no new signs of bleeding) and hemoglobin stabilization. Also, there was no additional requirement for blood transfusions. FVIII plasma activity shortly after infusion of rpFVIII was not available, but 24 hours later, the plasma activity was higher than predicted based on an assumed 8-hour half-life of exogenous rhFVIII (49% actual versus approximately 25% predicted), indicating an appropriate response and low likelihood of cross-reactive antibodies to rpFVIII. The patient was discharged to a skilled nursing facility the next day. High-dose prednisone (approximately 1 mg/kg = 80 mg/daily) was initiated on the first day of hospitalization. After discharge, the patient continued to receive high-dose prednisone for one month, which eradicated the inhibitory FVIII antibody. Thereafter, a slow taper was implemented (reduction by 10 mg/daily per week). Screening of plasma collected prior to administration of rpFVIII confirmed the absence of anti-porcine FVIII antibodies (proprietary test, Shire Pharmaceuticals).

**Figure 2:**
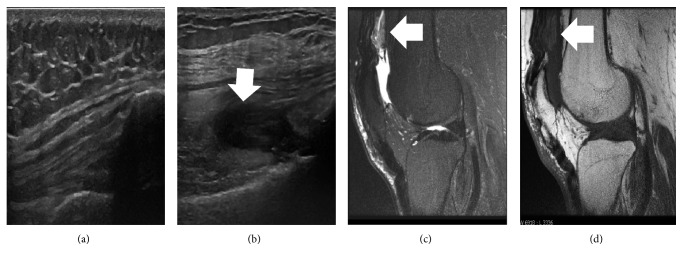
Point-of-care musculoskeletal ultrasound and magnetic resonance imaging of suprapatellar recesses in the knees. Longitudinal MSKUS image (a) of the unaffected knee was compared to the affected knee (b), which showed semicompressible hypoechoic contents (white arrow) in the suprapatellar recess. T2-weighted (c) and T1-weighted (d) MRI showed hyperintense (white arrows), corresponding to blood clots. MSKUS, musculoskeletal ultrasound; MRI, magnetic resonance imaging.

Fifteen weeks later, the patient presented at the UCSD Hemophilia and Thrombosis Treatment Center for right knee pain after a mechanical fall. Laboratory studies showed 116% FVIII activity with a FVIII inhibitor titer of <0.4 U. Point-of-care musculoskeletal ultrasound (POC MSKUS) of the right knee showed semicompressible hypoechoic contents in the suprapatellar, lateral, and medial recesses. Joint aspiration failed to withdraw fluid due to high resistance. These findings were consistent with coagulated blood in the joint space. Conventional MRI to assess other abnormalities did not reveal acute injury and also confirmed absence of an effusion (Figure 2). In conjunction with normal FVIII activity levels, this finding made acute hemarthrosis due to injury and lingering FVIII deficiency less likely although the exact time point of bleeding remained elusive. Also, the normalized FVIII activity level ruled out the development of clinically relevant, cross-reactive anti-porcine FVIII antibodies.

**Figure 3:**
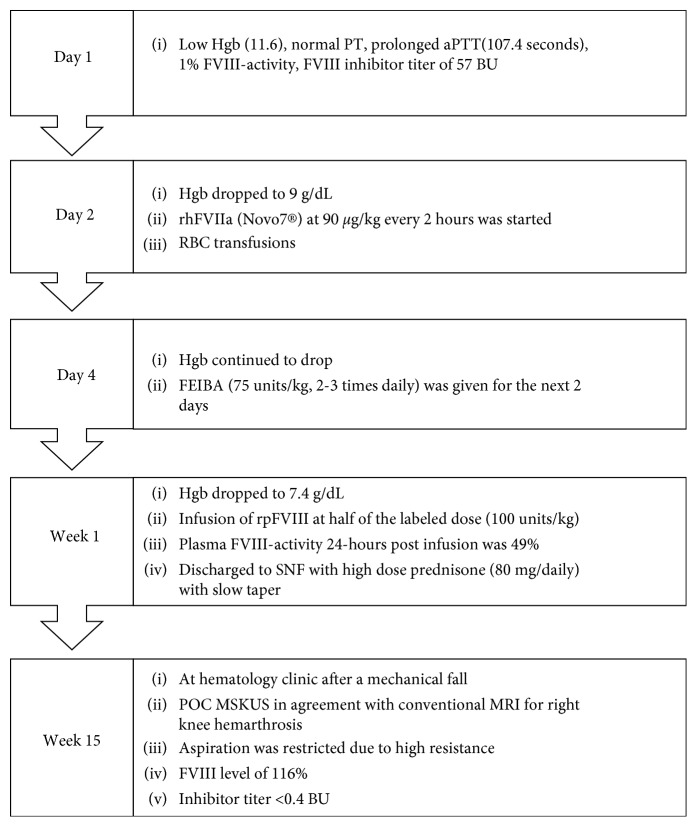
Timeline course of hospitalization stay and follow-up at the hematology clinic after a mechanical fall. High-dose corticosteroids were started on day 1 and tapered after one month, once eradication if the anti-FVIII antibody was achieved. Hgb, hemoglobin; PT, prothrombin time; aPTT, activated partial thromboplastin time; FVIII, Factor eight; BU, Bethesda units; rhFVIIa, activated recombinant human factor VII; RBC, red blood cell; FEIBA, Factor Eight Inhibitor Bypassing Activity; SNF, skilled nursing facility; POC MSKUS, point-of-care musculoskeletal ultrasound; MRI, magnetic resonance imaging.

Figure 3 depicts the timeline and course of the patient's management starting at diagnosis until inhibitor eradication.

This case demonstrates that early intervention with rpFVIII in AHA may be a valuable strategy. It also emphasizes the usefulness of POC MSKUS for management of musculoskeletal hemophilic bleeding.

## 2. Discussion

Acquired hemophilia A (AHA) is a rare and potentially fatal bleeding disorder. It is caused by autoantibodies against circulating coagulation Factor VIII (FVIII), leading to inhibition of Factor VIII binding to von Willebrand factor, activated factor IX, or negatively charged phospholipids [1]. The incidence of AHA ranges between 0.2 and 1.0 cases per one million per year [2]. Its prognosis is poor with a high mortality rate within the first several months after diagnosis (9–27%), due to comorbidities, advanced patient age, location, and severity of bleeding [3–5]. Management is often challenging due to delayed diagnosis with the suboptimal efficacy of FVIIa-based bypassing agents, resulting in fatal bleeding complications [6, 7].

The development of immune-mediated autoantibodies against FVIII in AHA is associated with a range of clinical disorders. Up to 50% of the cases are idiopathic [8, 9]. In the other half of the cases, development of autoantibodies is associated with solid or hematological neoplasms, autoimmune diseases, pregnancy, or medication effects (especially antibiotics and interferon) [10]. However, the mechanisms underlying the development of AHA remain poorly defined.

The management of AHA mainly consists of two components: efforts to achieve prompt hemostasis and autoantibody (“inhibitor”) eradication. The treatment for hemostatic control includes the use of plasma-derived or recombinant FVIIa bypassing agents, namely, Factor Eight Inhibitor Bypassing Activity (FEIBA®, Baxter Healthcare Corporation, Deerfield, IL, USA) or recombinant human (rh) FVIIa (Novo Seven; Novo Nordisk A/S, Bagsvaerd, Denmark). Both of these agents achieve hemostasis by generating thrombin in the absence of FVIII at the site of bleeding [11, 12]. However, they differ in their biochemical properties, composition, and pharmacokinetics. While FEIBA has a longer half-life of 4–7 hours, the half-life for rhFVIIa is approximately 2 hours [13, 14]. Neither of these agents have a reliable biomarker that correlates with clinical efficacy, and bleed containment cannot be achieved in all patients [10, 15]. In addition, both agents are associated with the risk of thromboembolic events, especially in patients with AHA who are frequently older and have comorbidities [16–18].

RpFVIII was approved by the Food and Drug Administration in 2014 for rescue treatment of severe bleeding in AHA patients. RpFVIII is a 1448 amino acid heterodimer with a molecular mass of 170 kDa. Its B-domain is partially deleted, allowing functional clotting FVIII while preventing inactivation by circulating human anti-FVIII antibodies. The recommended initial dose of rpFVIII is 200 U/kg. Therapeutic FVIII activity levels are generally achieved with goal FVIII levels >25% and maintained with intermittent rpFVIII administration until bleeding resolves. The advantages of this new therapy included no related serious adverse events, thrombotic events, allergic reactions, or thrombocytopenia in the registration study [10]. In addition, the level of FVIII can be directly monitored to evaluate its efficacy. However, its approval was granted based on a study of only 28 patients with little real-world experience available [19, 20].

From personal experience, it is postulated by hemophilia experts that rpFVIII may be more effective than FVIIa-based bypassing products. If correct, then rpFVIII might be the first agent used, but it is often not. Although rpFVIII shows excellent efficacy, it is rarely considered as the first-line treatment due to cost per dose. The cost of a single dose of rpFVIII per recommended label (200 U/kg) is approximately 10-fold higher than a single dose of FVIIa-based bypassing products. However, in some reports, restoration of hemostasis was obtained with lower doses of 100 U/kg rpFVIII [21, 22]; which was true in our patient. Based on this experience, we surmise that meaningful pharmacoeconomic savings may have been obtained if rpFVIII had been utilized front-line, not only sparing multiple doses of FVIIa-based bypassing agents but also shortening hospitalization.

Meanwhile, although it is not as common as in congenital hemophilia, hemarthrosis and muscle bleeds can occur in AHA [2]. Therefore, prompt diagnosis is critical to guide efficient medical management. Recently, we reported that the sensitivity of MSKUS to diagnose hemarthrosis surpasses the sensitivity of MRI, which is a widely accepted “reference standard” [23]. Using POC MSKUS allowed accurate and rapid detection of musculoskeletal bleeding in the clinic setting [24–26]. In this case, accurate diagnosis was critical to confidently diagnose the absence of hemarthrosis in the face of normalizing plasma FVIII levels, thereby preventing potentially prothrombotic interventions with hemostatic agents based on clinical perception alone. These observations illustrate the usefulness of POC MSKUS in detecting musculoskeletal bleeding not only in congenital hemophilia but also in AHA.

## 3. Summary and Conclusions

The patient represents an example of rare AHA, diagnosed in the setting of acute life-threatening bleeding that was successfully managed with a single low dose of rpFVIII after many doses of FVIIa-based bypassing agents. Treatment was followed by successful immune eradication of the inhibitory FVIII antibodies. This case highlights various new aspects in the management of AHA, including the usefulness of POC MSKUS for rapid detection of intra-articular and muscle bleeding. Most importantly though, it highlights the efficacy of rpFVIII, suggesting that front-line use rather than the use as a rescue agent may be a favorable strategy in AHA management.
